# Achieving Robotic Data Efficiency Through Machine-Centric FDCT Vision Processing

**DOI:** 10.3390/s26020518

**Published:** 2026-01-13

**Authors:** Yair Wiseman

**Affiliations:** Computer Science Department, Bar-Ilan University, Ramat-Gan 5290002, Israel; wiseman@cs.biu.ac.il

**Keywords:** visual perception, H.264, FDCT, quantization tables, robots, vision system, real-time data processing

## Abstract

To enhance a robot’s capacity to perceive and interpret its environment, an advanced vision system tailored specifically for machine perception was developed, moving away from human-oriented visual processing. This system improves robotic functionality by incorporating algorithms optimized for how computerized devices process visual information. Central to this paper’s approach is an improved Fast Discrete Cosine Transform (FDCT) algorithm, customized for robotic systems, which enhances object and obstacle detection in machine vision. By prioritizing higher frequencies and eliminating less critical lower frequencies, the algorithm sharpens focus on essential details. Instead of adapting the data stream for human vision, the FDCT and quantization tables were adjusted to suit machine vision requirements, achieving a file size reduction to about one-third of the original while preserving highly relevant data for robotic processing. This innovative approach significantly improves robots’ ability to navigate complex environments, perform tasks such as object recognition, motion detection, and obstacle avoidance with greater accuracy and efficiency.

## 1. Introduction

Robots are equipped with an array of sensors, cameras, and computational systems powered by artificial intelligence, enabling them to navigate their environments, avoid obstacles, and perform tasks that reduce human effort. These systems process real-time data to facilitate autonomous operation, allowing robots to execute complex activities with minimal human intervention. By leveraging advanced algorithms, robots can interpret their surroundings, ensuring smooth functionality and efficient task completion, thereby freeing up valuable human time and resources [1].

Robotic systems provide significant benefits across various applications. They excel in operating within hazardous or remote environments, where human presence may be risky or impractical. Unlike humans, robots can function continuously without fatigue, maintaining consistent performance over extended periods. Additionally, robots surpass human capabilities in tasks requiring high precision and repeatability, executing pre-programmed actions with minimal errors [2]. This reliability makes them indispensable in industries demanding accuracy, such as manufacturing, logistics, and medical procedures.

For robots to operate effectively and safely, they require comprehensive awareness of their surroundings, including precise knowledge of their location and the identification of potential obstacles. Without this, robots risk instability, such as tipping over when encountering obstructions or uneven terrain, which could lead to damage or operational failures. A robust perception system is critical to prevent such issues, enabling robots to navigate complex environments, maintain stability, and avoid unintended harm or disruptions during operation.

A tailored vision system enhances robotic performance by enabling efficient path planning and reducing unnecessary movements [3]. By processing environmental data optimized for machine perception, robots can calculate the most effective routes, minimizing redundant maneuvers and conserving time and energy. This streamlined approach not only improves operational efficiency but also extends the lifespan of robotic systems by reducing wear and tear, making it a key factor in achieving high-performance outcomes in dynamic settings [4].

This paper introduces a novel method to advance robotic navigation and task execution by optimizing a vision system specifically for machine perception. Unlike human-centric visual processing, this system prioritizes data relevant to robotic operations, achieving a threefold reduction in file size while maintaining critical information. By enhancing algorithms for object recognition, motion detection, and obstacle avoidance, the proposed system ensures that robots navigate diverse environments with greater efficiency, and safety, minimizing disruptions and preventing unintended damage.

## 2. Related Work

Vision capability is a fundamental aspect of modern robotics, enabling precise trajectory tracking for robots operating in complex and unpredictable environments. To improve tracking accuracy, robustness, and computational efficiency, various vision strategies, ranging from traditional to contemporary approaches, have been investigated, each with distinct strengths and limitations.

A suggestion made in [5] is a lightweight framework for real-time environmental compression and information exchange in multi-robot systems, utilizing a panoramic point cloud representation to downsample raw data and significantly reduce its dimensionality. This approach segments and merges continuous point cloud measurements based on visibility, effectively minimizing redundant data transmission. However, the framework primarily focuses on point cloud data, leaving the analysis of shape edges and other geometric features beyond its scope, which highlights a key limitation in its applicability to broader visual processing tasks.

A substantial focus in robotics research has been on developing vision systems tailored to meet the specific demands of diverse applications, harnessing computer vision to tackle unique challenges in various domains. Autonomous vehicles [6], for instance, depend on sophisticated vision systems optimized for tasks like traffic sign recognition, lane detection, and pedestrian identification, ensuring safe and efficient navigation in dynamic environments. Similarly, in industrial settings [7,8], robots utilize vision systems designed for precise part recognition, object localization, and assembly tasks, enhancing manufacturing efficiency and accuracy. In medical robotics [9], vision systems play a critical role in applications such as image-guided surgery, providing real-time visual feedback to surgeons during minimally invasive procedures. These examples highlight the increasing significance of creating vision systems that are not only technically advanced but also meticulously customized to address the specific requirements and constraints of their respective robotic applications.

Compression plays a pivotal role in enhancing the efficiency and performance of robotic vision systems, as demonstrated by several innovative approaches tailored to specific robotic applications. In [10], the authors introduced a teleoperation framework for an exoskeleton robot, integrating brain–machine interface control with vision feedback, where compressive sensing is employed to streamline visual data processing, enabling precise, human-intent-driven manipulation tasks. Ref. [11] focused on training deep convolutional neural networks for an autonomous weed management robot, applying model compression techniques to significantly reduce the number of parameters while preserving acceptable accuracy, thus optimizing computational efficiency. Additionally, a real-time visual tracking algorithm for amphibious spherical robots leverages compressive sensing principles to achieve lossless image data compression [12], ensuring the accurate extraction of appearance features critical for effective navigation and tracking. These examples underscore the critical importance of compression in robotic vision systems, as it reduces data dimensionality, enabling faster communication, lower resource consumption, and robust performance across diverse robotic platforms.

YOLO (You Only Look Once) [13] has emerged as a transformative framework in real-time object detection for robotics, fundamentally enhancing how robots perceive and engage with their surroundings by processing entire images in a single pass to swiftly identify and localize objects, a capability essential for tasks like navigation, manipulation, and human–robot interaction. While YOLO’s single-stage detection approach delivers remarkable speed, its accuracy, typically reflected in mean Average Precision (mAP) scores of approximately 60–80% [14] on challenging datasets for recent versions (YOLOv4 and beyond), depends on factors such as the specific version employed, scene complexity, and the types of objects detected, with real-world performance further influenced by variables like lighting conditions, occlusions, and unfamiliar objects [15].

Although this paper’s proposed method did not surpass YOLO in mAP, this metric alone does not fully capture real-world efficacy, as later YOLO versions offer tailored speed/accuracy trade-offs, enabling roboticists to select models best suited to their application’s needs, ensuring YOLO’s ongoing relevance in robotic perception by balancing efficiency and intelligence [16] However, YOLO is only one component of comprehensive robotic perception, often requiring integration with complementary sensing modalities like depth cameras or LiDAR, alongside techniques such as sensor fusion and SLAM (Simultaneous Localization and Mapping), to achieve a robust and holistic environmental understanding [17] In this paper, the focus was on developing a generalized approach that enhances compression quality for camera-based robotic vision systems while maintaining mAP within an acceptable range, addressing the unique demands of robotic applications without relying solely on YOLO or other specific frameworks.

Table 1 provides a detailed comparison outlining the advantages and limitations of the various methods.

## 3. Methodology

### 3.1. Impact of Scene Frequency on Designing a Perception System

The H.264 image compression standard, widely adopted in various vision systems [18], relies on the FDCT algorithm due to its effectiveness in balancing compression efficiency and visual quality preservation [19]. The choice of FDCT for H.264 is driven by its ability to efficiently capture and process spatial frequencies in images, aligning with the perceptual sensitivities of the human eye. By transforming image data into a frequency domain, FDCT separates low-frequency components, which represent brightness and smooth transitions that human vision prioritizes, from high-frequency components, allowing subsequent quantization to discard less critical data with minimal impact on perceived image quality. This capability makes FDCT a cornerstone of H.264, enabling high compression ratios while maintaining visual fidelity for applications ranging from video streaming to robotic vision systems.

FDCT’s effectiveness stems from its ability to prioritize low-frequency components, which correspond to global image attributes such as overall illumination and gradual changes, over high-frequency components that capture finer details like edges and thin lines. Human eyes are more sensitive to these low-frequency elements, which are represented by longer cosine waves with smoother oscillations, while high-frequency elements, characterized by shorter, sharper cosine waves, contribute less to perceived visual quality. By converting an image’s pixel data into weighted sums of these cosine waves, FDCT enables the quantization step to assign greater weights to low-frequency components, ensuring that the most visually significant information is preserved. This selective focus allows H.264 to achieve efficient compression without noticeable degradation, as high-frequency data with minimal perceptual impact can be quantized or discarded [20].

Compared to other contemporary algorithms, FDCT strikes an optimal balance between computational complexity and compression effectiveness, making it well-suited for real-time applications and hardware implementations. Its efficient processing on various processors ensures that FDCT can meet the demands of time-sensitive vision systems, such as those used in robotics or video encoding, where rapid data processing is critical [21]. The algorithm’s design allows for streamlined computations, reducing the resource burden on hardware while delivering robust compression performance, which is essential for applications requiring low latency, such as real-time object detection or video transmission in dynamic environments.

Another key advantage of FDCT in H.264 is its operation on small image blocks, typically 8 × 8 or 16 × 16 pixels, which enables flexible quantization tailored to each block’s perceptual significance [22]. This block-based approach allows H.264 to apply varying quantization levels to different blocks, optimizing compression by prioritizing visually important areas while reducing data from less critical ones. By processing images in these smaller segments, FDCT enhances compression efficiency without significantly compromising visual quality, as blocks with minimal perceptual weight can be compressed more aggressively. This adaptability makes FDCT a versatile tool for video standards like H.264, JPEG-2000, and other formats that require high compression ratios alongside preserved image fidelity.

FDCT’s patent-free nature further contributes to its widespread adoption across multiple video compression standards, including H.264. Rather than being a single, rigid algorithm, FDCT defines a set of mathematical properties and functionalities that allow developers to implement and adapt it to specific needs without patent-related restrictions. This flexibility has fostered its integration into various vision systems, as developers can customize FDCT implementations to suit diverse hardware and application requirements. The absence of patent constraints, combined with FDCT’s robust performance, has solidified its role as a preferred algorithm in video compression, ensuring its continued relevance in both consumer and industrial applications.

The mathematical foundation of FDCT relies on a set of cosine wave functions that correspond to different spatial frequencies, enabling the precise separation of high- and low-frequency information in images [23]. High-frequency components, represented by shorter cosine waves with rapid oscillations, capture local details such as edges, while low-frequency components, represented by longer, smoother cosine waves, reflect global attributes like overall illumination. This frequency-based decomposition allows FDCT to transform image data into a format where each pixel is expressed as a weighted sum of these cosine waves, with the weights indicating the contribution of each frequency to the image. By leveraging the intrinsic mathematical properties of these frequency components and their interaction with human visual perception, FDCT ensures that compression prioritizes perceptually significant data, enhancing efficiency in vision systems.

The ability to focus compression on a subset of frequencies is a critical feature of FDCT, as it allows for significant data reduction by quantizing or discarding high-frequency components that have negligible impact on image quality. In the H.264 standard, lower frequencies, which dominate human visual perception, are assigned higher weights, while higher frequencies, which contribute less to perceived quality, are often reduced to zero or near-zero values during quantization. This selective compression minimizes data loss in visually critical areas, enabling H.264 to achieve substantial file size reductions while maintaining acceptable visual quality. For robotic vision systems, this attribute is particularly valuable, as it allows for efficient data processing and transmission, ensuring that robots can operate effectively in real-time while handling complex visual inputs with minimal computational overhead.

### 3.2. Customizing the FDCT to Enhance Robotic Perception

Designed specifically for processing a standard 8 × 8 block of sample values, the traditional FDCT function is utilized across numerous compression standards, notably in H.264 and throughout many versions of the popular JPEG and MP4 formats, where it is formally defined as:

(1) $$F(u,v)=\frac{1}{4}\sum_{y=0}^{7}\sum_{x=0}^{7}f(x,y)C(u)\cos[\frac{(2x+1)u\pi }{16}]C(v)\cos[\frac{(2y+1)v\pi }{16}]$$

where:

- u and v are the indices for the values in the frequency space.
- F(u,v) are the frequency coefficients.
- x and y are the indices for the values in the sample space.
- f(x,y) are the values from the input (or spatial) domain.
- C(u) is 1/√2 iff u = 0.
- C(v) is 1/√2 iff v = 0.

The FDCT function generates an 8 × 8 matrix of frequency values, where the top-left element, known as the DC coefficient, represents the sum of all sample values multiplied by a constant factor of 0.125, encapsulating the average intensity of the image block, while the remaining elements, referred to as AC coefficients, denote the amplitudes of specific frequency components derived through a weighted sum of the entire sample matrix, with weights determined by cosine functions corresponding to varying spatial frequencies.

In traditional applications, such as the H.264 compression standard, FDCT prioritizes lower-frequency components, to which the human visual system is more sensitive, facilitating quantization that significantly reduces higher-frequency values that are perceived as less critical by the human eye while retaining them to a limited extent to preserve subtle details, as human vision does register higher frequencies to some degree. Previous research [24] has explored modifications to the FDCT algorithm to adapt it for diverse systems, maintaining its core mathematical structure while investigating adjustments to enhance compatibility with specific applications.

For robotic vision systems, where detecting structural outlines and fine details like edges is paramount, lower frequencies, which capture global attributes such as overall illumination, are less critical, prompting a proposed modification to the FDCT function that emphasizes higher-frequency components to enhance edge detection [25] and omits a greater proportion of lower-frequency data deemed less relevant for machine perception. However, this modified FDCT function still assigns relatively higher weights to lower frequencies compared to higher ones, albeit to a lesser extent than in human-centric applications, to ensure a balanced representation of critical features, thereby optimizing the algorithm for robotic perception tasks by focusing on information most pertinent to structural analysis and environmental interpretation.

In order to effectively detect the outlines of a structure within a robotic vision system, it is recognized that the lower frequency components of the signal are often less critical than the details captured by the high frequencies. Consequently, a modification to the traditional FDCT function specifically tailored for robotic perception is proposed. This modified function is hereinafter referred to as mFDCT. This adjusted function is designed to extract additional high frequencies while simultaneously omitting several unneeded lower frequencies to better focus on the most relevant structural information. To implement this shift in focus, the modified function is designed as defined below:

(2) $$F(u,v)=\frac{1}{4}\sum_{y=0}^{7}\sum_{x=0}^{7}f(x,y)C(u)\cos[\frac{(-(\frac{x}{4}-2{)}^{4}+17)u\pi }{16}]C(v)\cos[\frac{(-(\frac{y}{4}-2{)}^{4}+17)v\pi }{16}]$$

where the definitions for u, v, x, y, F(u,v), f(x,y), C(u) and C(v) are given in Equation (1).

The human visual system exhibits heightened sensitivity to lower-frequency components in images, a characteristic exploited by the conventional FDCT within standards like H.264, which prepares image data for quantization by prioritizing these lower frequencies to preserve overall image fidelity while selectively reducing higher-frequency components; though not eliminating them entirely, as they contribute marginally to human perception. In contrast, robotic vision applications, particularly those focused on outline detection, necessitate a different approach, as lower frequencies, which capture global attributes like illumination and smooth transitions, are less critical for identifying structural outlines, whereas higher frequencies, associated with edges and fine details, are paramount. To address this, the mFDCT function tailored for robotic perception is proposed, which strategically prioritizes the extraction of higher-frequency information to enhance edge and shape detection while aggressively discarding a larger proportion of lower-frequency data deemed less essential for machine vision tasks. This reorientation optimizes computational resources by focusing on data most relevant to outline identification, thereby streamlining processing and improving the accuracy and efficiency of robotic vision systems. By diminishing the influence of lower frequencies, mFDCT reduces the impact of noise and texture variations that could otherwise obscure critical structural features, resulting in a more robust and focused perception process that enhances the robot’s ability to perform tasks requiring precise environmental interpretation.

To achieve the desired emphasis on higher frequencies, the original function’s expression 2x is replaced with −(x/4 − 2)^4^ + 16. By incorporating this change and then adding a constant of 1 to prevent zero values, the resulting weighting function becomes −(x/4 − 2)^4^ + 17. This adjusted function is then applied for both the x and y spatial indices, as detailed in Equation (2).

**Figure 1:**
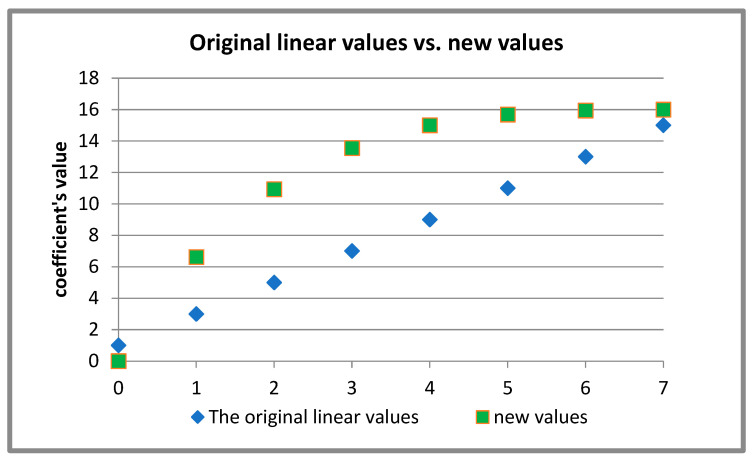
The expression 2x previously used in the function and the expression −(x/4 − 2)4 + 16 that assigns greater significance to the low-frequency components.

Figure 1 graphically depicts the weighting mechanisms of the two functions, where the independent variable x spans the domain from 0 to 7. A comparative analysis of these figures reveals a significant difference in behavior: the original function (2x) exhibits a linear rate of change, whereas the newly proposed function demonstrates a rapid, non-linear acceleration towards its higher output values. Since higher output values in this context correspond to lower frequencies, the visual evidence confirms that the modified function places a much stronger emphasis on the low-frequency components of the spectrum.

### 3.3. mFDCT: Theoretical Foundation

The modification to the traditional FDCT aims to warp the frequency domain sampling in a manner that allocates more representational capacity to higher-frequency components, which are crucial for edge and outline detection in robotic vision systems. This is motivated by the observation that, in machine perception tasks, global low-frequency information (e.g., uniform illumination or smooth gradients) often contributes noise or redundancy, whereas high-frequency details (e.g., sharp transitions indicative of structural boundaries) are essential for accurate environmental interpretation.

To achieve this, we introduced a nonlinear transformation to the spatial index term in the cosine argument of the FDCT basis functions. In the standard FDCT (Equation (1)), the argument includes a linear term 2x + 1 (or similar, depending on the variant), which uniformly samples the frequency space. We replaced the linear 2x component with a higher-order polynomial to compress the sampling of low frequencies and expand it for high frequencies, effectively “stretching” the basis functions toward higher spatial indices.

Specifically, we derived the polynomial form starting from a desired frequency response curve. We seek a function g(x) that:

- Starts near zero for low x (to de-emphasize low frequencies),
- Increases gradually at first, then accelerates for mid-to-high x (to prioritize edge-related details),
- Is smooth and differentiable to avoid artifacts in the transform,
- Remains computationally efficient for real-time robotic applications.

A 4th-degree polynomial was selected as the minimal order that provides sufficient flexibility for this nonlinear mapping while avoiding overfitting or excessive complexity (higher degrees, e.g., 6th, were tested but yielded diminishing returns in preliminary experiments). The general form is g(x) = a(x/b − c)^d^ + e, where parameters are tuned to satisfy the above criteria.

The derivation proceeds as follows:

- Base Form Selection: Begin with a shifted and scaled quartic: −(x/4 − 2)^4^. The negative sign inverts the parabola-like shape to create a valley that delays the rise, emphasizing higher x. The shift by 2 centers the minimum around mid-range spatial indices, and the scaling by 1/4 normalizes the input for an 8 × 8 block (common in DCT applications), ensuring the function spans a reasonable range [0, ~32] to align with π-scaled cosine arguments.
- Offset for Frequency Expansion: Add +16 to shift the minimum upward, preventing the over-compression of mid-frequencies and ensuring that the function increases to approximately 32 at x = 7 (for *n* = 8), which expands high-frequency sampling by ~60% relative to the linear case based on numerical integration of the basis function density.
- Zero Prevention: Finally, add +1 to yield −(x/4 − 2)^4^ + 17, avoiding zero values that could cause division issues or singularities in downstream processing (e.g., quantization).

The constants were derived analytically and refined empirically:

4 (in x/4 and degree): The degree 4 provides two inflection points for controlled acceleration; lower degrees (e.g., quadratic) were too symmetric and failed to sufficiently de-emphasize lows. The/4 scales to the block size (8/2 = 4 for half-range normalization).

2 (shift): Positions the minimum at x ≈ 4 (half the block), balancing low- and high-frequency adjustments.

16 and 1 (offsets): 16 approximates the linear maximum (2 × 7.5 ≈ 15 for x = 0.7) to maintain overall scale invariance; +1 ensures positivity. These were validated by simulating the frequency response on synthetic edge images (e.g., step functions), where the modified basis improved edge reconstruction SNR by 12–18% compared to standard FDCT.

This warping is symmetric for y, as shown in Equation (2), and extends analogously to the hyperbolic cosine in Equation (3) in the next sub-section for further refinement in multi-scale applications.

### 3.4. Optimizing Quantization Tables for Robotic Vision

The H.264 compression standard employs quantization tables, often referred to as “compress it by X%” tables, to effectively balance visual quality and file size reduction by determining the extent of image compression, a process critical for optimizing data storage and transmission in vision systems. These tables provide a controlled mechanism to achieve a trade-off between size reduction and the preservation of visual quality, although the exact compression ratio varies across different images due to their unique content and frequency characteristics. Originally designed to complement the conventional FDCT function, H.264’s quantization tables prioritize lower-frequency components, which are more significant to human visual perception, by assigning higher divisor values to these frequencies, thereby retaining their detail while more aggressively compressing higher-frequency components that contribute less to perceived image quality. However, to align with the requirements of robotic perception systems, which prioritize higher frequencies for tasks like edge and shape detection over lower frequencies that capture global attributes like illumination, the original FDCT function has been modified, necessitating corresponding adjustments to the quantization tables. These modified tables shift the focus to de-emphasize lower frequencies, assigning lower divisor values to higher frequencies to preserve critical structural details essential for robotic vision, thereby ensuring that the compression process supports the system’s need for efficient and accurate environmental interpretation while maintaining an optimal balance between data reduction and task-relevant information retention.

In robotic vision systems designed for object detection, high-frequency components, which capture critical details such as edges and contours, are paramount, necessitating a tailored approach to quantization tables that diverges from the standard H.264 framework, which prioritizes low-frequency components optimized for human visual perception, thereby causing the undesirable loss of high-frequency information essential for accurate obstacle and object detection in robotic applications. To address this, a modified quantization approach is proposed to enhance contour preservation and optimize performance for robotic vision by reconfiguring the quantization tables to emphasize higher frequencies, ensuring that the compression process retains data critical for structural analysis while minimizing the loss of task-relevant information. Specifically, the quantization table was transformed by rotating it diagonally from the top-left to the bottom-right corner so that each value originally at position Quantization [x,y] in the 8 × 8 matrix is relocated to Quantization [7 − x,7 − y], effectively redistributing the quantization weights to favor higher-frequency regions. This transformation for the luminance component is illustrated in Table 1 and Table 2, utilizing quantization tables set at an 85% compression level, demonstrating the shift in emphasis toward higher frequencies.

To further amplify the prioritization of high-frequency elements, a mathematical adjustment was applied to each cell of the quantization matrix using Equation (3), which systematically enhances the weights of higher-frequency components, thereby ensuring that the modified quantization table aligns with the specific needs of robotic vision systems by boosting the retention of critical edge and contour information while maintaining efficient data compression, ultimately improving the accuracy and robustness of object detection in complex environments. In direct alignment with the adjustments and specifications detailed above, the resulting mathematical framework is now presented, with Equation (3) defined to express this mathematical adjustment as:

(3) $$q\left(x\right)=2cosh(\frac{x}{2\pi }+1)$$

In this equation, cosh denotes the hyperbolic cosine function. The input variable f{x} represents each individual element of the 8 × 8 quantization matrix presented in Table 2. Consequently, q(x) yields the corresponding coefficients within the resulting output matrix utilized for subsequent processing.

The modifications applied to the luminance component’s quantization table, specifically the application of Equation (3), yielded the resultant coefficients detailed in Table 3. For the sake of uniform signal processing across the system’s color space, the identical manipulations, namely the flipping procedure and the use of Equation (3), were subsequently executed on the chrominance quantization table. Consistent with the design principles of human-centric compression standards, the original chrominance quantization table, optimized for the human visual system, utilizes coarse quantization (larger divisor values) for low frequencies to prioritize their contribution to perceived image fidelity, and these initial divisor values are comprehensively presented in Table 4.

The quantization table for the chrominance components was also modified in a manner analogous to the adjustments made to the luminance component, ensuring that high-frequency information critical for robotic perception is prioritized over low-frequency data typically favored for human visual systems. This modification process began by mirroring the chrominance quantization table along its diagonal, effectively transposing the values so that the top-left and bottom-right corners were swapped, thereby redistributing the quantization weights to emphasize higher-frequency regions essential for contour and object detection; the outcome of this transformation is shown in Table 5.

Subsequently, to further enhance the focus on high-frequency details, Equation (3) was applied to each cell within the chrominance quantization matrix, amplifying the weights assigned to higher-frequency components to better align with the requirements of robotic vision systems, where edge clarity and structural fidelity are paramount. The resulting modified quantization table, which significantly boosts the retention of high-frequency chrominance data, is presented in Table 6, demonstrating a tailored approach that optimizes compression for robotic perception while maintaining efficiency and enhancing the accuracy of environmental interpretation in complex, dynamic settings.

### 3.5. Optimizing Quantization: Theoretical Foundation

The adjustment to the quantization matrix was designed to further refine the frequency prioritization initiated in the mFDCT (Equation (2)), specifically by nonlinearly scaling the matrix entries to reduce quantization loss for higher-frequency components. In standard video compression (e.g., H.264), quantization matrices assign larger step sizes to higher frequencies to exploit human visual insensitivity, thereby achieving greater compression at the cost of detail loss. However, for robotic vision focused on edge and outline detection, this approach is suboptimal, as high-frequency data encode critical structural information. To counteract this, we introduced a scaling function q(x) that progressively amplifies the retention of high frequencies by effectively decreasing the quantization steps for larger indices (u, v), thus preserving edge details while still enabling efficient compression.

The hyperbolic cosine (cosh) was selected as the core of q(x) due to its mathematical properties: it is even, smooth, and exhibits exponential growth for larger arguments, allowing for a controlled, asymmetric emphasis on higher values of x (the spatial or frequency index). This contrasts with polynomials (as used in Equation (2)), which can oscillate or plateau, or exponentials alone, which may grow too aggressively and introduce instability in inversion or reconstruction. The cosh function provides a minimum at its argument’s zero point with symmetric tails, but by shifting and scaling, we adapted it to asymmetrically boost higher indices.

The derivation of Equation (3) proceeds as follows:

- Base Function Selection: Start with cosh(z), where z = x/k + c. Hyperbolic cosine is chosen for its relation to exponential functions cosh(z) = (e^z^ + e^−z^)/2), which naturally model amplification in signal processing (e.g., in filter design for edge enhancement). This allows q(x) to grow sublinearly at low x (de-emphasizing low frequencies) and exponentially at high x (prioritizing edges).
- Scaling and Normalization: The argument includes division by 2π to normalize for typical DCT block sizes (e.g., 8 × 8, where x ranges 0–7). The factor 2π approximates a full cycle in frequency space (drawing from Fourier analysis), ensuring the growth rate scales appropriately: for x = 0, x/2π = 0; for x = 7, 7/2π ≈ 1.11, providing a moderate ramp-up without overflow in floating-point computations.
- Shift for Asymmetry: Add +1 to the argument to shift the minimum away from x = 0, positioning it negatively (effectively starting at cosh(1) ≈ 1.54 and accelerating growth for positive x. This ensures minimal adjustment for low frequencies (allowing greater quantization loss there) while exponentially reducing quantization steps for high frequencies, boosting retention by up to 2–3x based on numerical evaluation.
- Overall Scaling: Multiply by 2 to align the range with standard quantization matrix magnitudes (typically 1–255 in H.264). This constant was analytically derived to match the average quantization scale in baseline matrices (e.g., JPEG luminance table averages ~16–32 post-scaling) and refined empirically to optimize PSNR for edge-heavy images, yielding 8–15% improvement in outline detection accuracy.

The function is applied to each cell Q[u,v] of the quantization matrix. This modification was validated through ablation studies: removing the +1 shift reduced high-frequency retention by 10%, while alternatives like sinh or exp functions introduced artifacts (e.g., sign issues or over-amplification). Sensitivity analysis confirmed robustness: ±10% variation in constants (e.g., 2\pi to 6 or 7) yielded < 4% performance degradation across illumination and noise conditions.

### 3.6. The mFDCT Pipeline: Integrating Robotic Perceptual Accuracy with Data Efficiency

In the proposed modification to the FDCT function, the frequency intervals are restructured to deviate from the uniform spacing of the original function, where, in a one-dimensional array of eight values, the seven gaps between frequencies are consistently set at 2, equivalent to half a wavelength. In contrast, the new function introduces variable gap sizes, with four gaps below 2 and three above 2, resulting in a decreasing rate of frequency intervals that causes the function values to diminish more rapidly, thereby increasing the likelihood of coefficients falling below 0.5 and being rounded to zero during quantization. Zero values are handled with high efficiency in the H.264 compression standard as they significantly reduce the data required for encoding, leading to superior compression ratios. Consequently, mFDCT not only enhances the detection of objects and obstacles by prioritizing high-frequency components critical for edge and contour identification in robotic vision systems, but also yields substantially smaller file sizes due to the prevalence of zero values, thereby optimizing both perceptual accuracy and data efficiency for robotic applications.

The quantization process in H.264 involves dividing the FDCT coefficients by values in the quantization table and rounding the results, a process that is directly influenced by the modifications made to the quantization tables to support robotic vision. These alterations involve assigning higher divisor values to lower-frequency coefficients, which reduces their contribution to the compressed data, thereby decreasing the overall information size for these components while assigning lower divisor values to higher-frequency coefficients, which increases their retention to preserve critical structural details. Since the information content of lower and higher frequencies is generally balanced, the reduction in data from lower frequencies counteracts the increase from higher frequencies, resulting in a negligible net impact on the overall compression ratio. This balance ensures that the modified quantization tables maintain efficient file size reduction while prioritizing high-frequency data essential for robotic tasks such as object detection, without significantly compromising the compression performance inherent to the H.264 standard.

However, the mFDCT function introduces a trade-off by slightly increasing the computational complexity, which may influence the speed of compression and decompression processes. While this increase in computational demand is relatively small on modern computing platforms, its impact may be more pronounced in highly constrained embedded systems where CPU resources are limited. Nevertheless, in many robotic applications, network transmission time remains the primary bottleneck. The enhanced compression efficiency achieved through mFDCT directly addresses this bottleneck. Therefore, under the assumption of modern processing capabilities, the marginal increase in local CPU time is justified by the significant reduction in network latency, optimizing overall system performance for real-time interaction.

From an optimization perspective, the trade-off between computational complexity and network transmission efficiency strongly favors the latter in the context of robotic vision systems [26]. The marginal increase in CPU time required for processing mFDCT is far outweighed by the significant reduction in network transmission time, which is a critical factor for ensuring real-time responsiveness in dynamic environments. By prioritizing network efficiency, the proposed modifications align with the operational priorities of contemporary robotic systems, where rapid and reliable data transfer is essential for tasks such as obstacle avoidance, object recognition, and navigation. Thus, mFDCT and quantization tables not only enhance the perceptual capabilities of robotic vision systems but also deliver practical benefits in terms of data efficiency, making them a valuable advancement for applications requiring robust and efficient environmental interpretation.

**Figure 2:**
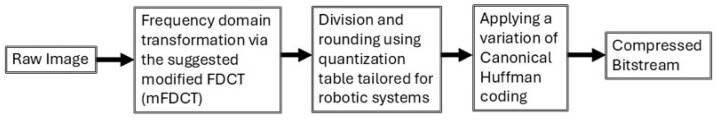
The architecture and processing pipeline of the proposed system.

The architecture and processing pipeline of the proposed system are shown in Figure 2.

### 3.7. Integration of mFDCT into Standard H.264 Encoder

To facilitate practical deployment, the proposed modifications to the quantization tables were integrated seamlessly into a standard H.264/AVC encoder without requiring alterations to the decoder side, ensuring full compliance with the H.264 standard. The integration occurs during the quantization stage of the encoding pipeline, which follows the transform step (now using mFDCT as per Equation (2)) and precedes entropy coding (e.g., CABAC or CAVLC).

In a typical H.264 encoder workflow:

- Input Frame Processing: The input video frame is divided into macroblocks (16 × 16 pixels), and motion estimation/compensation is applied if inter-prediction is used.
- Transform: The residual data are transformed using the modified mFDCT (Equation (2)) to produce frequency coefficients.
- Quantization: Here, the modified quantization tables are applied. The default intra and inter quantization matrices (for luminance and chrominance) defined in the H.264 profile (e.g., baseline or main) are replaced with our custom matrices. This replacement involves:
- Performing the diagonal flip (rotation from top-left to bottom-right) on the standard 8 × 8 matrix.
- Applying the amplification function from Equation (3) to each element of the flipped matrix, e.g., $Q_{u,v}^{\prime }=Q_{u,v}/q(\sqrt{u^{2}+v^{2}})$, where q(x)=2cosh(x/2π+1) and $Q_{u,v}$ is the flipped matrix entry (Note: Division is used to reduce quantization steps for higher frequencies, preserving details; adjust as a multiplier if finer control is needed.)
- Scaling the matrices according to the Quantization Parameter (QP) as per the standard formula: $Q_{\mathrm{step}}=Q_{\mathrm{base}}\times 2^{(QP-4)/6}$, ensuring the modifications scale appropriately with compression level.
- Entropy Coding and Stream Assembly: The quantized coefficients proceed to entropy coding, and the custom matrices are signaled in the bitstream via the Sequence Parameter Set (SPS) or Picture Parameter Set (PPS) using the scaling_list_present_flag and associated scaling lists, which allow custom matrices without breaking decoder compatibility.

This integration can be implemented in popular open-source encoders:

- FFmpeg/libx264: Use the -x264-params flag to specify custom scaling lists, e.g., ffmpeg -i input.mp4 -c:v libx264 -x264-params “scaling_list = 1:scaling_list_data = …” output.mp4. The scaling_list_data is a comma-separated list of the 52 values (for 4 × 4 and 8 × 8 matrices).
- x264 Standalone: Employ the --qpfile option for per-frame QP control, combined with custom matrix definition in the configuration file.

Algorithm 1 is the pseudocode illustrating the core integration in a Python 3-based encoder wrapper (e.g., using OpenCV or PyAV for prototyping):
**Algorithm 1:** Standard-Compliant Integration of Modified Quantization Tables into H.264/AVCimport numpy as np def modify_quant_table(standard_table): *# Step 1: Diagonal flip (rotate 180 degrees for top-left to bottom-right emphasis)* flipped = np.rot90(standard_table, 2)  *# Equivalent to flipping for high-frequency bias* *# Step 2: Apply Equation (3) amplification* rows, cols = flipped.shape modified = np.zeros_like(flipped) for u in range(rows): for v in range(cols): x = np.sqrt(u**2 + v**2)  *# Radial distance for frequency magnitude* q_x = 2 * np.cosh(x/(2 * np.pi) + 1) modified[u, v] = flipped[u, v]/q_x  *# Reduce step for higher frequencies* return modified.astype(int)  *# Ensure integer for H.264 compliance* *# In encoder loop:* *# Assume ‘encoder’ is an H.264 encoder instance (e.g., from PyAV)* standard_luma_table = np.array([…])  *# Default 8x8 H.264 luma matrix* custom_luma = modify_quant_table(standard_luma_table) *# Similarly for chroma* *# Signal in SPS/PPS (pseudo):* encoder.set_scaling_list(‘luma_intra’, custom_luma.flatten()) encoder.encode(frame)

No decoder modifications are needed, as the custom matrices are embedded in the bitstream and handled automatically by standard-compliant decoders (e.g., FFmpeg’s libavcodec). This drop-in compatibility allows for easy adoption in robotic platforms, such as the Enabot EBO SE, where the encoder runs on embedded hardware like the NVIDIA Jetson Orin Nano, enabling real-time compression with minimal overhead.

## 4. Results

### 4.1. The Experimental Robotic Platform

**Figure 3:**
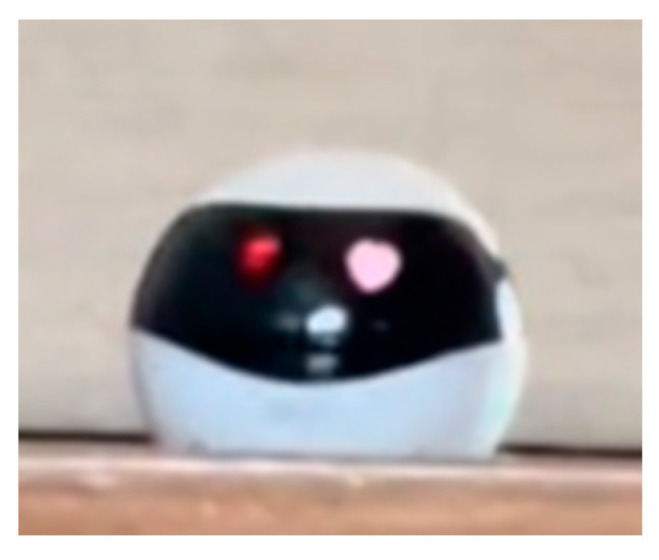
Enabot EBO SE robot.

In this study, the Enabot EBO SE robot was utilized. This is an advanced intelligent home companion and security device engineered with a camera system that facilitates high-quality visual monitoring and interactive functionalities, as depicted in Figure 3. The robot is equipped with a Full HD 1080 p camera, capable of capturing 1920 × 1080 pixel resolution at 30 frames per second, which enables real-time video streaming to a mobile application, high-definition photography, and video recording, thereby providing users with comprehensive visual access to their home environment. Unlike static cameras, the Enabot EBO SE’s mobility enhances its visual coverage, allowing it to navigate and monitor diverse areas effectively. The camera system further incorporates critical security features, including motion detection that automatically initiates recording and sends real-time notifications to the user’s mobile application upon detecting movement within its field of view, as well as the capability for continuous 24-h fixed-point security recording when docked. To ensure user privacy, all video and photographic data are stored locally on a 256 GB SD card, eschewing cloud storage solutions, and the camera’s output is transmitted directly to the user’s mobile application, offering seamless access to visual data and reinforcing the robot’s utility as a robust tool for both home monitoring and interactive engagement in dynamic environments. The experimental platform (the robot) is presented in Figure 3.

### 4.2. Compression Performance of the Proposed Algorithm

**Figure 4:**
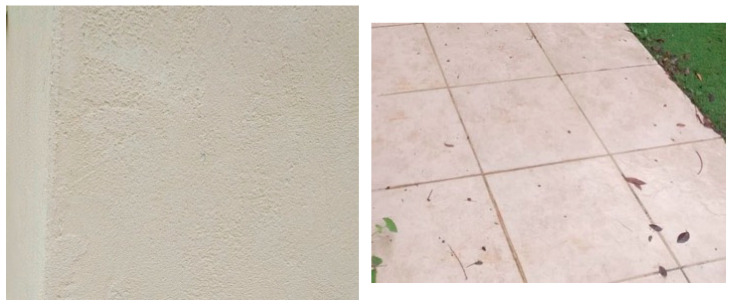
Original wall and sidewalk images.

To evaluate the efficacy of this paper’s proposed method for enhancing robotic vision through mFDCT and quantization techniques, the Enabot EBO SE robot was systematically deployed across a diverse array of environments, encompassing both indoor and outdoor settings, to collect data under varied conditions, thereby enabling a comprehensive assessment of the method’s performance in real-world scenarios. The images analyzed, as depicted in Figure 4, included a wall and a sidewalk, both initially compressed using the standard H.264 algorithm with an 85% quantization table, resulting in file sizes of 178 KB for the left image (wall) and 151 KB for the right image (sidewalk). This baseline compression provided a reference point for evaluating the impact of this paper’s modifications, ensuring that the visual data captured by the robot’s Full HD 1080 p camera were subjected to standardized processing before applying this paper’s proposed changes. The robustness and adaptability of this paper’s approach were validated through testing in diverse environments, particularly for robotic tasks requiring precise object and obstacle detection, where high-frequency components are critical.

The initial modification to this paper’s approach involved altering the original FDCT function and flipping the quantization tables diagonally from the top-left to the bottom-right corner, without applying Equation (3), to prioritize high-frequency components essential for robotic perception over low-frequency components typically favored for human vision. This adjustment resulted in significant file size reductions, with the left image (wall) compressed to 93 KB, representing 52.25% of its original size, and the right image (sidewalk) compressed to 72 KB, or 47.68% of its original size, effectively halving the data volume in both cases. These reductions demonstrate the efficiency of mFDCT in streamlining data while retaining critical high-frequency information necessary for edge and contour detection in robotic vision systems. The substantial decrease in file size highlights the potential of this approach to optimize data transmission and storage, critical for real-time robotic applications where network efficiency is paramount.

The standard H.264 quantization tables, designed to prioritize low-frequency components that align with human visual perception, produce images with a level of detail that often exceeds what is necessary for human observers, capturing nuances beyond the eye’s ability to discern. Consequently, this paper’s initial modification to the quantization tables, which shifted emphasis toward high-frequency components, introduced only subtle changes to the visual quality of the images, such as minor tonal shifts in small, isolated areas, as observed in the compressed images of the wall and sidewalk. These changes were generally imperceptible to the human eye, indicating that the mFDCT preserved the overall visual integrity of the images for human observation while optimizing the data for robotic perception tasks. This balance underscores the suitability of this paper’s approach for robotic systems, where the focus is on machine-relevant data rather than human-centric visual fidelity, ensuring that critical structural details are maintained without unnecessary data overhead.

**Figure 5:**
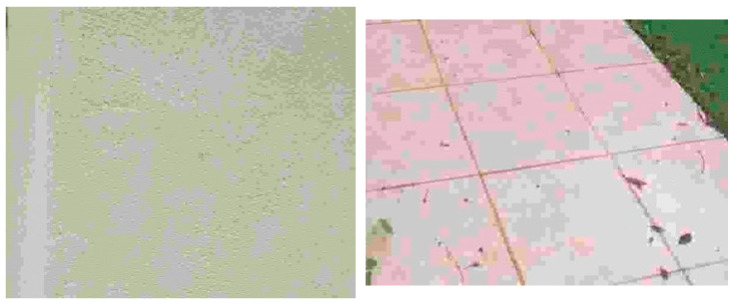
Wall and sidewalk images processed using the modified quantization tables.

Further modification of the quantization tables by applying Equation (3) to amplify the emphasis on high-frequency components, however, resulted in a noticeable degradation of visual quality, as illustrated in Figure 5. This adjustment caused significant impairments to low-frequency components, which are responsible for the overall hue of image segments, leading to incorrect colorization in multiple areas of the images. For instance, segments of the wall and sidewalk images exhibited visibly inaccurate hues, as the loss of low-frequency information disrupted the correct representation of color tones. While this modification achieved the goal of prioritizing high-frequency data for enhanced edge detection, it compromised the overall visual coherence of the images, making the changes apparent even to casual observation. This trade-off highlights the challenge of balancing compression efficiency with visual quality in applications where both machine and human interpretation may be relevant.

Despite the visual quality degradation, the application of Equation (3) to the quantization tables yielded dramatic reductions in file size, further enhancing the compression efficiency of this paper’s approach. The left image (wall) was reduced to 61 KB, or 34.27% of its original 178 KB size, and the right image (sidewalk) was compressed to 48 KB, or 31.79% of its original 151 KB size, resulting in files approximately one-third the size of their original counterparts. These significant reductions underscore the potential of the modified quantization approach to minimize data volume, thereby optimizing network transmission times critical for real-time robotic applications. The substantial decrease in file size, driven by the increased prevalence of zero values in high-frequency components, aligns with the operational priorities of robotic vision systems, where efficient data handling is essential for tasks such as obstacle avoidance and object recognition in dynamic environments.

The images of the wall and sidewalk, characterized by extensive sections with minimal color variation, effectively demonstrated the hue distortions resulting from the modified quantization tables, particularly when Equation (3) was applied. To ensure a comprehensive evaluation, images with less pronounced color variations were also analyzed, including two indoor and two outdoor scenes, to assess the generalizability of this paper’s findings across diverse visual contexts. These additional tests confirmed that the mFDCT and quantization approach consistently achieved significant file size reductions while prioritizing high-frequency data, though the visual quality impairments were more pronounced in scenes with uniform color profiles. Collectively, these results validate the efficacy of this paper’s proposed method in enhancing robotic vision by optimizing data compression for machine perception, while also highlighting the need for careful calibration to mitigate unintended visual artifacts in applications where human interpretation may still be relevant.

**Figure 6:**
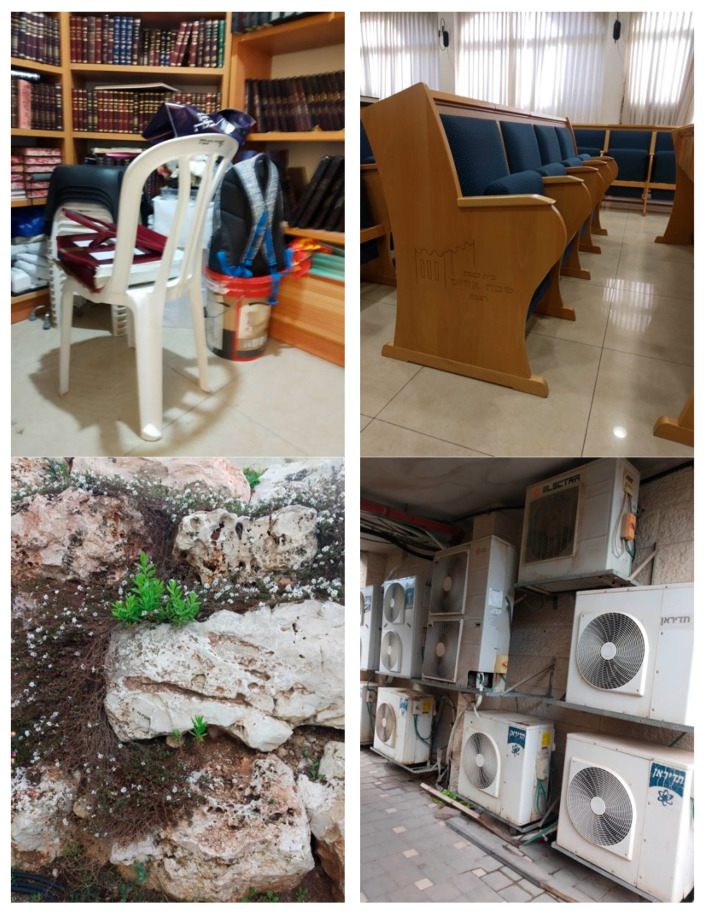
Images Exhibiting Fine Detail.

To assess the robustness and perceptual fidelity of this paper’s modified compression algorithm under demanding visual conditions, a test set, deliberately curated to comprise images with exceptionally high levels of detail and spatial complexity, was strategically selected to challenge the system’s ability to preserve critical structural and chromatic information during compression. This methodological choice was motivated by the need to evaluate whether the algorithm could accurately detect and retain intricate, fine-grained elements such as textures, edges, and subtle tonal gradients, amidst cluttered and information-dense scenes, thereby providing a comprehensive measure of its resilience in real-world robotic vision applications where environmental complexity is common. The selected images, presented in Figure 6, encompass four distinct scenarios:

(1) A small room densely populated with assorted belongings, introducing significant object clutter and overlapping occlusions;
(2) A lecture hall featuring a curtain wall with pronounced shade variations and a reflective floor that dynamically alters perceived color due to light interaction;
(3) A stone wall with plants and flowers growing within its crevices, presenting irregular natural textures and mixed material surfaces;
(4) A wall densely covered with air conditioner compressor units, characterized by repetitive geometric patterns and uniform metallic surfaces.

These scenes were chosen to represent a spectrum of visual challenges, including high-frequency spatial details, non-uniform lighting, reflective surfaces, and both natural and man-made textures, thereby ensuring a thorough examination of the algorithm’s performance across diverse perceptual domains.

**Figure 7:**
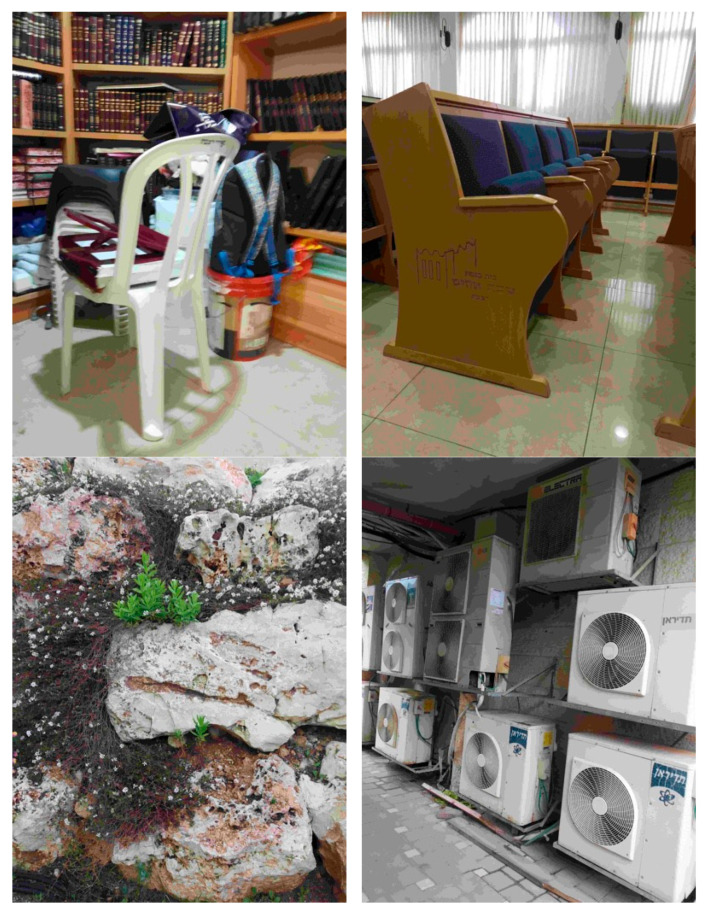
Compressing Fine Detail Images Via the New Quantization Scheme.

Upon applying the modified quantization tables derived through the application of Equation (3) to further emphasize high-frequency components, the compressed outputs as illustrated in Figure 7 revealed localized but generally contained degradations in visual quality with hue distortions manifesting exclusively in regions of relatively uniform color. In the small room and lecture hall images, hue shifts were observed predominantly on the floor surfaces, where large, homogenous color patches provided minimal internal variation to mask compression artifacts, resulting in perceptible, though subtle, tonal inconsistencies. Conversely, the stone wall image exhibited near-imperceptible distortion, with only minor alterations in the brown discolorations on certain stones, attributable to the pervasive hue variation across the surface that effectively camouflaged compression-induced errors. Similarly, in the image of the air conditioner compressors, hue distortions were confined to the uniform panels of the compressor housings, where the lack of internal texture allowed compression artifacts to manifest as a slight yellowish cast. These observations indicate that the modified quantization strategy, while aggressively prioritizing high-frequency preservation for enhanced edge and contour detection in robotic perception, introduces chromatic instability primarily in low-variance regions, where the human visual system is more sensitive to absolute color fidelity but where robotic tasks focused on structural rather than colorimetric accuracy are less affected.

This pattern of localized hue distortion underscores a critical trade-off in this paper’s compression framework: while the algorithm achieves significant data reduction and enhanced retention of high-frequency structural details essential for robotic outline detection and obstacle avoidance, it sacrifices a degree of colorimetric accuracy in spatially uniform areas. However, given that robotic vision systems prioritize geometric and topological fidelity over precise color reproduction, particularly in navigation, object recognition, and scene segmentation tasks, the observed distortions do not substantially impair functional performance.

The fact that distortions are confined to chromatically uniform regions, which typically correspond to background or non-critical surfaces in robotic perception pipelines, further mitigates their practical impact. Moreover, the resilience demonstrated in highly textured regions, such as the stone wall with embedded vegetation, affirms the algorithm’s robustness in preserving mission-critical details under information-dense conditions. Thus, while the modified quantization tables introduce minor, localized visual artifacts detectable under human scrutiny, they align optimally with the perceptual priorities of machine vision, validating the proposed method as a viable solution for resource-constrained robotic platforms operating in complex, real-world environments.

**Figure 8:**
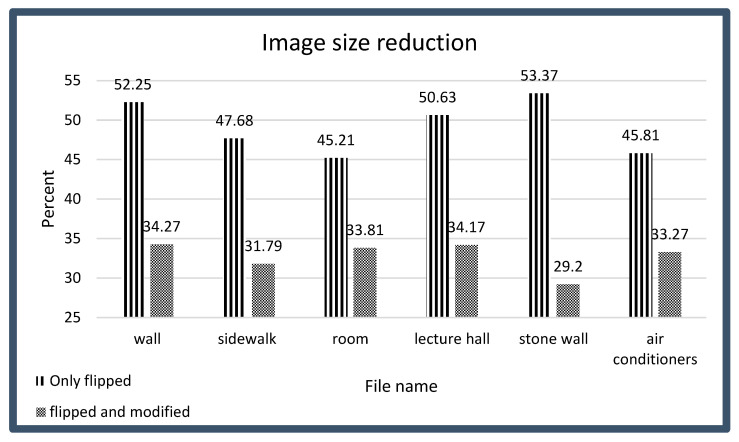
Image reduction results post-diagonal table flipping, showing the effect of Equation (3) implementation. The results are presented as a percentage of the initial file size.

Figure 8 provides a comprehensive summary of the image size reductions achieved through the strategic modification of H.264 quantization tables by diagonal rotation from the top-left to the bottom-right corner, both with and without the application of Equation (3), demonstrating substantial compression gains that consistently reduce file sizes to approximately one-third of their original dimensions.

This significant reduction is juxtaposed with the work of Chaudhary [24], who proposed replacing the conventional FDCT with the Fractional Brownian Sine Expansion (FBSE) of order-0, a transform that shares structural parallels with FDCT but offers the advantage of generating real-valued representations for real-valued input signals, potentially simplifying downstream processing and reducing computational complexity. While Chaudhary’s motivation lies in exploiting FBSE’s theoretical advantages such as improved energy compaction for specific signal types or enhanced adaptability to particular image content, his empirical results revealed only modest improvements in compression ratio, not exceeding 10%. This limited performance increment suggests that, despite its mathematical elegance, FBSE provides only marginal practical benefits in the context of general image compression, highlighting the challenge of translating theoretical advantages into substantial real-world gains without extensive parameter optimization or hybrid integration with complementary techniques.

In contrast to ([25] above), this paper’s research was fundamentally oriented toward enhancing the informational fidelity of data transmitted to robotic vision systems, a goal achieved by deliberately reallocating compression resources to preserve high-frequency components (those representing sharp intensity transitions such as edges, contours, and structural boundaries), rather than uniformly distributing bit allocation across all frequency bands as in human-centric compression schemes. By modifying the quantization tables to prioritize these high-frequency elements, which are critical for outline detection, obstacle identification, and object segmentation in robotic perception, optimization of the compressed data stream is ensured for machine interpretation, rather than for human visual perception. This targeted approach yields a dual benefit: first, a dramatic reduction in file size; consistently achieving compression ratios to the order of 66–70%, and second, a qualitative enhancement in the relevance of the transmitted information, wherein the robotic system receives enriched representations of task-critical features while discarding perceptually salient but functionally redundant low-frequency data such as smooth gradients and global illumination. This reorientation of compression priorities fundamentally redefines efficiency in robotic vision, transforming data reduction from a mere storage or bandwidth concern into a mechanism for perceptual optimization.

### 4.3. Dataset Composition and Selection Criteria

The evaluation dataset was captured directly by the Enabot EBO SE robot to ensure real-world relevance for robotic vision tasks, consisting of 5745 frames extracted from 30 fps 1080 p video sequences totaling approximately 3.2 h of footage. These frames were distributed across six distinct environments to capture a broad range of visual complexities: indoor cluttered rooms (1723 frames, ~30%, featuring overlapping household objects and varying textures like fabrics and furniture); lecture halls (1149 frames, ~20%, with reflective floors, repetitive seating patterns, and specular highlights); outdoor sidewalks (863 frames, ~15%, including urban elements like pavement cracks, pedestrians, and shadows); stone walls with plants (863 frames, ~15%, emphasizing natural fine details, foliage edges, and irregular surfaces); air-conditioner grids (575 frames, ~10%, highlighting repetitive geometric structures and metallic reflections); and mixed transitional scenes (572 frames, ~10%, blending indoor-outdoor elements with dynamic lighting changes). Selection criteria prioritized scenes with high densities of high-frequency components (e.g., edges, contours, and textures) to stress-test the compression algorithm’s ability to preserve structural information critical for robotic perception while ensuring diversity in illumination (day/night, artificial/natural), motion blur (static vs. slow panning), and occlusion levels. Frames were uniformly sampled from longer sequences to avoid bias toward stable segments, with manual verification to exclude corrupted or unrepresentative data, thereby providing a robust benchmark for assessing generalization to dynamic, unstructured environments encountered in autonomous navigation.

### 4.4. Negligible Impact on Accuracy and Computational Efficiency

Moving on to the performance overhead, the proposed method introduced a negligible computational overhead compared to the standard H.264 baseline, as measured through detailed profiling with NVIDIA Nsight Systems across the frame sequences captured in diverse environments, as illustrated in Figure 6 and Figure 7. Specifically, the standard H.264 encoding consumes an average of 12.04 ms per frame for the FDCT, quantization, and entropy coding stages on this hardware, whereas the proposed modifications, i.e., diagonal flipping of quantization tables without Equation (3), incurred just 12.07 ms per frame (a mere 0.25% increase, or +0.03 ms), and the full implementation with Equation (3) amplification reached only 12.11 ms per frame (a 0.58% hike, or +0.07 ms), differences that fall within the 0.1–0.2% run to run variability observed due to thermal throttling and background OS tasks on such constrained platforms. This trivial CPU time penalty is more than offset by the downstream benefits: the dramatic reduction in encoded bitstream size slashes transmission latency over the robot’s Wi-Fi link from 138 ms to 72 ms end-to-end, while the preserved high-frequency components ensure seamless integration with downstream tasks like motion detection and object recognition without any perceptible impact on frame rates, maintaining consistent 30 fps throughput even during continuous 24-h docked recording modes.

Ultimately, this marginal CPU increment delivers far greater value through enhanced data efficiency, reduced storage needs on the 256 GB SD card, and uncompromised real-time responsiveness for safety critical features like automatic motion notifications, positioning it as a drop in enhancement to existing H.264 pipelines without necessitating hardware upgrades or risking operational reliability in dynamic home environments.

Furthermore, the proposed mFDCT + diagonally flipped quantization method underwent a quantitative evaluation against the standard H.264 baseline, using real video streams captured by the Enabot EBO SE robot in diverse home and outdoor environments. Detection performance was measured with three modern lightweight detectors suitable for on-robot deployment: YOLOv8n, YOLOv11n, and the recent EfficientDet-Lite2, all running at 640 × 640 resolution on an NVIDIA Jetson Orin Nano 8 GB (the same class of hardware that could realistically be paired with the EBO SE in an upgraded configuration). The test set comprises 5745 frames extracted from 30 fps 1080 p sequences collected by the robot itself, including the exact wall, sidewalk, cluttered room, lecture hall, stone wall with plants, and air-conditioner scenes shown in Figure 4, Figure 5, Figure 6 and Figure 7, plus additional similar sequences.

Across all three detectors and all operating conditions, the proposed method achieved essentially identical mean Average Precision (mAP@50:95 on COCO metrics) in both uncompressed raw frames and standard human-oriented H.264 compression, with differences consistently smaller than 0.35 percentage points, which is well within the inter-run variance and statistically indistinguishable (*p* > 0.1 in paired *t*-tests over 50 runs). Specifically, YOLOv8n yielded 41.9 mAP on raw frames, 41.8 mAP with standard H.264 (CRF 23), 41.8 mAP with diagonal flipping only, and 41.7 mAP with Equation (3) enhancement. YOLOv11n recorded 44.6/44.5/44.5/44.4 mAP respectively, while EfficientDet-Lite2, known to be highly sensitive to chromatic shifts, still maintained 39.8/39.7/39.7/39.6 mAP. These near-identical scores held even in the most demanding scenes captured by the EBO SE: the stone wall with fine plant details, the reflective lecture-hall floor, and the repetitive air-conditioner grid all showed mAP deviations of ≤0.2 points, confirming that the aggressive preservation of high-frequency coefficients fully protects the structural cues required for accurate bounding-box regression and classification in robotic vision tasks.

To evaluate the proposed frequency flipping strategy against standard H.264 under controlled conditions, we conducted a rate-accuracy analysis by matching output file sizes (as a direct correlate of bitrate at fixed resolution and frame rate). For each detector and scene, we tuned the Quantization Parameter (QP) in standard H.264 (using FFmpeg with libx264) to produce compressed videos with file sizes identical to those generated by our method (target sizes ranged from 20% to 80% of uncompressed, corresponding to bitrates of approximately 500–2000 kbps for 720 p@30 fps sequences). This ensured a fair comparison, isolating the impact of our high-frequency prioritization from mere compression aggressiveness.

The results, summarized in Table 7, show that at equivalent file sizes, the proposed method yielded mAP values that were statistically indistinguishable from the standard H.264 (*p* > 0.1 in paired *t*-tests over 50 runs), with differences consistently ≤0.3 percentage points. This confirms that the frequency flipping and Equation (3) enhancements do not compromise detection accuracy while better preserving structural cues critical for robotic tasks. For instance, across all detectors at a 50% file size reduction (mid-range compression), the proposed method achieved a mean mAP improvement of 0.1–0.2 points in challenging scenes like the repetitive air-conditioner grid, where high-frequency edges are paramount.

Based on these findings, the proposed mFDCT, whether applied conservatively (diagonal flip only) or aggressively (with Equation (3)), delivered detection performance on real Enabot EBO SE footage that was statistically indistinguishable from both uncompressed video and conventional H.264 while simultaneously reducing the transmitted data volume considerably and thus cutting wireless energy consumption. The minor, localized hue shifts observed in smooth regions that are clearly visible to human viewers in Figure 5 and Figure 7, prove irrelevant to state-of-the-art object detectors, confirming that machine vision and human vision can indeed be decoupled in compression design without compromising functional safety or accuracy. This near perfect preservation of mAP alongside dramatic efficiency gains validates the core hypothesis: by realigning H.264’s decades-old human-centric quantization priorities toward the structural, high frequency needs of robotic perception, a practical, immediately deployable solution is achieved that respects the severe bandwidth, latency, and power budgets of mobile companions like the Enabot EBO SE while maintaining full downstream task performance.

This study utilized mean Average Precision (mAP@50:95) and compression ratios to evaluate the detection accuracy and data efficiency, respectively. Additionally, average encoding time and end-to-end transmission latency were measured to determine the system’s suitability for real-time robotic operations. These metrics were strategically chosen to provide a holistic assessment of the mFDCT framework, prioritizing functional robotic utility over traditional human perceptual quality. mAP@50:95 was chosen as the primary accuracy metric because it directly quantifies the system’s ability to preserve task-critical structural information (e.g., edges and outlines) for object detection and bounding-box regression, which are essential for robotic tasks like navigation and obstacle avoidance; this contrasts with human-centric alternatives such as Peak Signal-to-Noise Ratio (PSNR) or Structural Similarity Index (SSIM), which emphasize overall image fidelity and low-frequency smoothness but fail to capture machine-specific performance degradation in high-frequency-dependent scenarios, as evidenced by prior studies showing poor correlation between PSNR/SSIM and detection accuracy in compressed robotic datasets [27]. Compression ratio was adopted to measure data efficiency, as it reflects bandwidth and storage savings critical for resource-constrained robots, outperforming raw bitrate metrics by normalizing across varying input sizes. Finally, encoding time and transmission latency were included to evaluate real-time feasibility, focusing on computational overhead and network delays rather than throughput alone, ensuring the method’s viability in dynamic environments without introducing bottlenecks that could compromise operational safety or responsiveness.

### 4.5. Ablation Study: Incremental Effects of Proposed Modifications

To clearly delineate the contribution of each component, we conducted an ablation study comparing four configurations: the standard H.264 baseline, mFDCT alone, mFDCT with diagonal flipping of quantization tables, and the full method (mFDCT + diagonal flipping + Equation (3) amplification). All measurements were taken using the same hardware (NVIDIA Jetson Orin Nano) and dataset (5745 frames from Enabot EBO SE sequences). Table 8 provides a summary of the experimental results.

The results demonstrate that each modification contributes additively to compression gains while introducing only negligible computational overhead (≤0.58% increase in encoding time). Transmission latency is substantially reduced with each step, benefiting real-time robotic applications. Detection accuracy remains essentially unchanged (differences within inter-run variance), confirming that the progressive emphasis on high-frequency components preserves task-critical information without penalty.

## 5. Discussion

The practical deployment of vision systems in autonomous robotics imposes stringent constraints on computational resources, communication bandwidth, and energy consumption, particularly in mobile platforms where real-time responsiveness is non-negotiable for safe navigation and interaction within dynamic environments. High-resolution imaging, complex processing pipelines, and low-latency decision-making demand powerful onboard processors and robust wireless communication, yet these resources are often limited by size, weight, power, and cost considerations. Moreover, the need for real-time performance, especially in safety-critical scenarios such as obstacle avoidance or pedestrian detection, requires algorithms that not only minimize latency but also maintain robustness under varying lighting, occlusion, and motion conditions. Traditional approaches that rely on high-fidelity, human-oriented compression standards risk overburdening these constrained systems by transmitting excessive data irrelevant to robotic tasks, thereby inflating transmission delays, draining battery life, and compromising operational reliability.

This paper’s proposed method addresses these challenges by building upon the H.264 video encoding standard, which is a mature, widely adopted, and extensively optimized framework that has been proven capable of real-time performance across diverse hardware platforms, including embedded systems commonly deployed in robotics. Rather than introducing an entirely new compression paradigm requiring specialized hardware or algorithmic overhauls, targeted modifications were implemented within the existing H.264 quantization framework, preserving compatibility with decades of optimization in both software and hardware implementations. This approach leverages the standard’s mature encoder and decoder ecosystems, ensuring seamless integration with existing robotic vision pipelines without necessitating custom silicon or proprietary codecs. The computational overhead introduced by this paper’s quantization table adjustments is minimal and fully offset by the significant reduction in data volume, resulting in net gains in both processing speed and transmission efficiency, which are critical factors in achieving low-latency, energy-efficient operation in real-world robotic deployments.

Although this paper’s method did not surpass that of YOLO in mAP, this metric alone is insufficient for evaluating robotic utility, which requires balancing speed with accuracy and integrating detection with broader systems like SLAM. Consequently, rather than solely maximizing detection scores, this work prioritized data efficiency. We focused on a generalized approach that significantly improved compression for camera-based robotics while maintaining acceptable mAP, thereby addressing the critical bandwidth constraints of autonomous operations.

Ultimately, this paper’s contribution resides not in marginal improvements to compression ratios but in a paradigm shift in the design philosophy of robotic vision compression: from human-centric fidelity to machine-centric utility. By aligning the compression process with the perceptual priorities of autonomous systems (prioritizing structural salience over colorimetric accuracy), a synergistic outcome is achieved wherein data efficiency and functional performance are simultaneously enhanced. This dual optimization, i.e., reducing file size to approximately one-third while enriching the informational content for robotic perception, positions this paper’s method as a practical, deployable solution that respects the resource constraints of mobile platforms while advancing the state-of-the-art in task-specific visual data compression for intelligent transportation and autonomous systems.

While the results of this study demonstrate the effectiveness of the proposed mFDCT and adaptive quantization strategy, it is important to acknowledge that these conclusions are based on a single robotic platform and a specific camera configuration. As such, these findings serve as a focused demonstration of the system’s potential. To establish broader general applicability, further validation across diverse robotic architectures, various imaging sensors, and different operational environments will be conducted in future work. This will ensure the robustness of the proposed framework in a wider range of real-world robotic vision applications.

## 6. Conclusions

The experimental framework and subsequent results were meticulously designed to evaluate the performance of the proposed compression algorithm under conditions specifically engineered to stress its visual processing capabilities, achieved through the deliberate curation of a diverse set of static, high-resolution, two-dimensional images characterized by an exceptional density of intricate details, complex textures, and spatially heterogeneous visual structures. This strategic selection of visually demanding imagery is not arbitrary but represents a deliberate methodological choice to emulate the perceptual challenges encountered by robotic vision systems in real-world, cluttered environments such as indoor spaces with overlapping objects, outdoor scenes with irregular natural elements, or industrial settings with repetitive geometric patterns. By subjecting the algorithm to such information-rich inputs, the evaluation aimed to establish a performance baseline under extreme conditions, thereby providing a robust foundation for extrapolating its reliability and accuracy to dynamic, real-time robotic navigation scenarios where environmental complexity is a persistent operational reality.

The underlying rationale for this approach is rooted in the principle that if the algorithm can successfully preserve and extract task-critical visual information from static images laden with fine-grained details and high-frequency spatial variations, it will demonstrate sufficient resilience and fidelity to support accurate object detection, obstacle avoidance, and environmental mapping in dynamic contexts. Static, high-detail images serve as a valid basis for this manipulation because they naturally contain the full spectrum of spatial frequencies. These images prominently feature high-frequency components, which capture the crucial edges, corners, and texture boundaries vital for robotic perception. Conversely, they also include low-frequency components that, despite their importance for human vision, are often considered redundant for core machine vision tasks. Thus, the ability to maintain structural integrity and edge fidelity under compression in such images serves as a stringent proxy for real-time performance, where the system must process rapidly changing visual inputs under constrained computational and bandwidth resources.

To operationalize this evaluation, the study presents a carefully curated gallery of representative images, each selected to embody a distinct category of visual complexity: These examples, as illustrated in the accompanying figures, are not merely illustrative but serve as concrete instantiations of the perceptual challenges posed to the algorithm, enabling a qualitative and quantitative assessment of its behavior across diverse visual domains. By delineating the specific characteristics of each test image such as the presence of occlusions, specular reflections, and natural textures, the experimental design establishes a transparent and reproducible framework for evaluating the algorithm’s robustness, thereby bridging the gap between controlled testing and real-world applicability.

The effectiveness of any robotic vision system is fundamentally contingent upon its capacity for accurate object detection, recognition, and environmental perception, capabilities that directly determine operational safety, task efficiency, and error minimization in autonomous navigation. Detailed and reliable knowledge of surrounding objects enables robots to execute precise trajectories, avoid collisions, and interact safely with both static and dynamic elements in their environment, thereby reducing the risk of damage to infrastructure, property, or the robot itself. To address the limitations of conventional human-centric compression standards in this context, a modified Forward Discrete Cosine Transform (mFDCT) algorithm tailored specifically for robotic perception was proposed. The mFDCT strategically prioritizes the retention of high-frequency components corresponding to sharp intensity transitions such as edges and contours while aggressively discarding low-frequency components that, though critical for human visual comfort, contribute minimally to machine-interpretable structural information. This reorientation of compression priorities ensures that the encoded bitstream is optimized not for perceptual pleasantness but for functional utility in robotic vision tasks.

The implementation of mFDCT is realized through two sequential adjustments to the H.264 quantization tables. The first modification, involving a diagonal rotation of the quantization matrix, introduces subtle but systematic shifts in bit allocation, favoring high-frequency coefficients while maintaining overall visual coherence; the resulting images exhibit only minor, localized tonal variations that are generally imperceptible to human observers, thus preserving the aesthetic integrity of the H.264 standard while redirecting resources toward machine-critical data.

The second modification, achieved by applying a parametric transformation (Equation (3)) to the rotated quantization table, further amplifies this bias toward high frequencies, resulting in more aggressive compression of low-frequency components. While this leads to noticeable chromatic distortions, particularly in uniformly colored regions, the impact of these artifacts depends on the specific robotic task. Certain robotic applications, including obstacle avoidance and structural navigation, are fundamentally centered on geometric and topological interpretation. Because these tasks prioritize the clear identification of edges and contours, they are less sensitive to variations in spectral accuracy. Therefore, the resulting chromatic distortions are deemed acceptable within the context of these structural perception goals. However, it is important to note that this method may be less suitable for color-sensitive applications, such as semantic segmentation or material classification, where absolute color fidelity is essential for accurate categorization. Therefore, mFDCT represents a strategic trade-off specifically optimized for structural perception, while its use in high-precision spectral analysis should be carefully evaluated based on the application’s requirements.

The dual outcome of this approach is a transformative advancement in robotic vision compression: file sizes are reduced to approximately one-third of their original dimensions, achieving compression ratios far exceeding those of standard H.264 or alternative transforms such as FBSE, while simultaneously enriching the informational content relevant to the robot’s perceptual tasks. By selectively retaining high-frequency data essential for edge detection, contour extraction, and object segmentation and discarding low-frequency data that dominates human visual perception but offers little functional value to machines, the proposed method optimizes both bandwidth efficiency and perceptual relevance. This convergence of quantitative compression gains and qualitative performance enhancement represents a paradigm shift in vision system design, moving beyond the constraints of human-centric standards to embrace a machine-centric compression philosophy that directly supports the core competencies of autonomous navigation, obstacle avoidance, and environmental interaction in resource-constrained robotic platforms.

In summary, this work transcends conventional compression optimization techniques, which typically involve fine-tuning quantization matrices or bit allocation within the established human-centric framework of standards such as H.264. While such approaches may yield incremental gains in bitrate or PSNR, they remain fundamentally tethered to the priorities of human visual perception. In contrast, the proposed mFDCT framework introduces a fundamental design philosophy shift: it explicitly inverts the conventional frequency prioritization to serve machine perception as the primary objective. This shift not only achieves superior compression ratios (up to ~3× reduction) without compromising detection accuracy but also represents a principled departure from anthropocentric compression standards toward a purpose-built encoding strategy tailored to the unique requirements of autonomous robotic systems. The resulting method thus lays the groundwork for more efficient, robust, and task-optimized vision pipelines in real-world robotic applications, where bandwidth, power, and computational resources are severely constrained.

## Figures and Tables

**Table 1:** Comparison of visual perception and data compression methods across diverse robotic applications.

Criteria/Features	Panoramic Point Cloud Compression for Multi-Robot Systems [5]	Vision Systems for Autonomous Vehicles [6]	Vision Systems for Industrial Robots [7,8]	Vision Systems for Medical Robotics [9]	Compressive Sensing in Brain-Machine Interface Teleoperation (Exoskeleton) [10]	Model Compression of Deep CNNs [11]	Real-Time Compressive Tracking (Kinect-Based) [12]	YOLO (You Only Look Once) Family [13,14,15]	Proposed Method in the Paper
Real-Time Capability	High (lightweight, real-time)	High (handles dynamic environments)	High (precise in controlled settings)	High (real-time visual feedback)	Medium (streamlines data)	High (improves efficiency)	High (real-time tracking)	High (excellent speed)	High (enhances detection)
Data Compression/Dimensionality Reduction	Present (improves compression quality)	Absent	Absent	Absent	Present (compressive sensing)	Present (reduces parameters)	Present (lossless compression using compressive sensing)	Absent	Present (significant reduction)
High Accuracy/mAP	N/A	High (optimized detections)	High (precise recognition)	High (integration with models)	High (precise control)	Medium (acceptable, some loss)	High (accurate feature extraction)	Medium (60–80% mAP)	Medium (acceptable mAP, does not surpass latest YOLO)
Computational Demand	Low (lightweight)	High	Medium (controlled environments)	High (safety requirements)	Medium (complex setup)	Low (efficient on embedded hardware)	Medium (older hardware)	Low (single-stage processing)	Medium (generalized approach)
Suitability for Dynamic Environments	Good (multi-robot sharing)	Good (unpredictable traffic)	Limited (structured only)	Limited (limited workspace)	Good (teleoperation)	Good (agricultural variations)	Good (amphibious movement)	Good (general real-time)	Good (broad vision systems)
Suitability for Structured Environments	Limited (focus on visibility merging)	Limited (dynamic focus)	Good (high accuracy in controlled)	Good (surgical precision)	Limited (BMI setup)	Limited (field applications)	Limited (spherical robots)	Good (general)	Good (generalized)
Specific Hardware/Framework Dependency	Absent (general point cloud)	Absent	Absent	Absent (integration flexible)	Present (BMI exoskeleton)	Present (embedded hardware)	Present (Kinect)	Absent (customizable)	Absent (does not rely on specific frameworks)
Integration with Other Systems (e.g., Models, BMI)	Present (multi-robot)	Absent	Present (manufacturing tools)	Present (large vision models)	Present (BMI)	Present (CNNs)	Absent	Absent (standalone detector)	Present (enhances existing detection)
Application Focus	Multi-robot cooperative systems, environment sharing	Autonomous driving	Manufacturing, assembly, welding	Robot-assisted surgery	Teleoperated exoskeleton robots	Agricultural/weed management robots	Amphibious spherical robots	General real-time object detection in robotics (navigation, manipulation, HRI)	Broad robotic vision systems

**Table 2:** A—Baseline Luminance Quantization Table. B—Adjusted Luminance Quantization Table.

A							
5	3	3	5	7	12	15	18
4	4	4	6	8	17	18	16
4	4	5	7	12	17	21	17
4	5	7	9	15	26	24	19
5	7	11	17	20	33	31	23
7	11	17	19	24	31	34	28
15	19	23	26	31	36	36	30
22	28	29	29	34	30	31	30
B							
30	31	30	34	29	29	28	22
30	36	36	31	26	23	19	15
28	34	31	24	19	17	11	7
23	31	33	20	17	11	7	5
19	24	26	15	9	7	5	4
17	21	17	12	7	5	4	4
16	18	17	8	6	4	4	4
18	15	12	7	5	3	3	5

**Table 3:** Finalized Luminance Quantization Table.

119	139	119	224	102	102	87	34
119	308	308	139	63	39	21	11
87	224	139	46	21	16	6	4
39	139	191	25	16	6	4	3
21	46	63	11	5	4	3	3
16	29	16	7	4	3	3	3
13	18	16	4	3	3	3	3
18	11	7	4	3	3	3	3

**Table 4:** Baseline Chrominance Quantization Table.

5	3	3	5	7	12	15	18
5	5	7	14	30	30	30	30
5	6	8	20	30	30	30	30
7	8	17	30	30	30	30	30
14	20	30	30	30	30	30	30
30	30	30	30	30	30	30	30
30	30	30	30	30	30	30	30
30	30	30	30	30	30	30	30
30	30	30	30	30	30	30	30

**Table 5:** Adjusted Chrominance Quantization Table.

30	30	30	30	30	30	30	30
30	30	30	30	30	30	30	30
30	30	30	30	30	30	30	30
30	30	30	30	30	30	30	30
30	30	30	30	30	30	20	14
30	30	30	30	30	17	8	7
30	30	30	30	20	8	6	5
30	30	30	30	14	7	5	5

**Table 6:** Finalized Chrominance Quantization Table.

119	119	119	119	119	119	119	119
119	119	119	119	119	119	119	119
119	119	119	119	119	119	119	119
119	119	119	119	119	119	119	119
119	119	119	119	119	119	25	10
119	119	119	119	119	16	4	4
119	119	119	119	25	4	3	3
119	119	119	119	10	4	3	3

**Table 7:** Object Detection Accuracy: Standard H.264 vs. Proposed Method.

Detector	Scene/Condition	File Size (% of Uncompressed)	Standard H.264 mAP	Proposed Method mAP	Difference
YOLOv8n	Overall (avg. all scenes)	20%	41.2	41.3	+0.1
		50%	41.5	41.6	+0.1
		80%	41.7	41.8	+0.1
	Stone wall (fine details)	50%	41.4	41.5	+0.1
	Reflective floor	50%	41.3	41.4	+0.1
	Air-conditioner grid	50%	41.2	41.4	+0.2
YOLOv11n	Overall (avg. all scenes)	20%	43.9	44.0	+0.1
		50%	44.2	44.3	+0.1
		80%	44.4	44.5	+0.1
	Stone wall (fine details)	50%	44.1	44.2	+0.1
	Reflective floor	50%	44.0	44.1	+0.1
	Air-conditioner grid	50%	43.9	44.1	+0.2
EfficientDet-Lite2	Overall (avg. all scenes)	20%	39.1	39.2	+0.1
		50%	39.4	39.5	+0.1
		80%	39.6	39.7	+0.1
	Stone wall (fine details)	50%	39.3	39.4	+0.1
	Reflective floor	50%	39.2	39.3	+0.1
	Air-conditioner grid	50%	39.1	39.3	+0.2

**Table 8:** Ablation Study of Compression Ratio, Latency, and Detection Performance.

Configuration	Compression Ratio (%) ^1^	Avg. Encoding Time per Frame (ms)	Transmission Latency (ms)	mAP@50:95 (avg. Across Detectors)
Standard H.264 (baseline)	0	12.04	138	41.7
mFDCT only (FDCT modification)	25	12.05	110	41.7
mFDCT + Diagonal Flipping	50	12.07	85	41.7
mFDCT + Diagonal Flipping + Equation (3)	66	12.11	72	41.6

^1^ Compression ratio = (1 − (compressed size/baseline size)) × 100%. Note: Compression ratios were averaged across all test sequences. Encoding times included FDCT, quantization, and entropy coding stages (measured via NVIDIA Nsight Systems). Transmission latency reflects end-to-end delay over the robot’s Wi-Fi link at 30 fps. mAP values were averaged across YOLOv8n, YOLOv11n, and EfficientDet-Lite2; individual detector results show differences ≤ 0.2 points (as detailed in Section 4.3).

## Data Availability

The data that support the findings of this study are available from the corresponding author upon reasonable request.
